# The Cause of Cholera

**Published:** 1867-06

**Authors:** 


### The Cause of Cholera.

In a memoir addressed to the Paris Academy of Medicine, M. Daumé announces the discovery of a cholera animalcule. The memoir has been referred to the committee having charge of the Bréant prize to be awarded to the person who discovers a cure for the modern pestilence. It is possible that Asiatic cholera may be caused by the presence of an organism; but from the rapidity of the progress of this disease, it seems more reasonable to infer that this organism belongs to the vegetable class, resembling minute mycelium, which often increase with most fearful rapidity. Still we are without proof to show that inorganic matter cannot assume such baneful form, by the process of diffusion through common air, as to carry into the lungs a poison which may soon disintegrate the blood and paralyze the nervous system. It has, fortunately, been clearly demonstrated that the progress of this unknown enemy through the favorite haunts—the cities—can be prevented by cleanliness, the use of disinfectants, and strict observance of sanitary regulations.

